# CADM1 Controls Actin Cytoskeleton Assembly and Regulates Extracellular Matrix Adhesion in Human Mast Cells

**DOI:** 10.1371/journal.pone.0085980

**Published:** 2014-01-22

**Authors:** Elena P. Moiseeva, Kees R. Straatman, Mark L. Leyland, Peter Bradding

**Affiliations:** 1 Institute for Lung Health, Dept. of Infection, Immunity and Inflammation, University of Leicester, Leicester, United Kingdom; 2 Centre for Core Biotechnology Services, University of Leicester, Leicester, United Kingdom; 3 Department of Biochemistry, University of Leicester, Leicester, United Kingdom; Hungarian Academy of Sciences, Hungary

## Abstract

CADM1 is a major receptor for the adhesion of mast cells (MCs) to fibroblasts, human airway smooth muscle cells (HASMCs) and neurons. It also regulates E-cadherin and alpha6beta4 integrin in other cell types. Here we investigated a role for CADM1 in MC adhesion to both cells and extracellular matrix (ECM). Downregulation of CADM1 in the human MC line HMC-1 resulted not only in reduced adhesion to HASMCs, but also reduced adhesion to their ECM. Time-course studies in the presence of EDTA to inhibit integrins demonstrated that CADM1 provided fast initial adhesion to HASMCs and assisted with slower adhesion to ECM. CADM1 downregulation, but not antibody-dependent CADM1 inhibition, reduced MC adhesion to ECM, suggesting indirect regulation of ECM adhesion. To investigate potential mechanisms, phosphotyrosine signalling and polymerisation of actin filaments, essential for integrin-mediated adhesion, were examined. Modulation of CADM1 expression positively correlated with surface KIT levels and polymerisation of cortical F-actin in HMC-1 cells. It also influenced phosphotyrosine signalling and KIT tyrosine autophosphorylation. CADM1 accounted for 46% of surface KIT levels and 31% of F-actin in HMC-1 cells. CADM1 downregulation resulted in elongation of cortical actin filaments in both HMC-1 cells and human lung MCs and increased cell rigidity of HMC-1 cells. Collectively these data suggest that CADM1 is a key adhesion receptor, which regulates MC net adhesion, both directly through CADM1-dependent adhesion, and indirectly through the regulation of other adhesion receptors. The latter is likely to occur via docking of KIT and polymerisation of cortical F-actin. Here we propose a stepwise model of adhesion with CADM1 as a driving force for net MC adhesion.

## Introduction

Mast cells (MCs) are highly specialised secretory cells, which serve as a first-line of defence against infections and environmental toxins. They are involved in the induction of an immune response to various pathogens and are also an integral part of the adaptive immune response [1], [2]. MCs are notorious for their roles in allergy, asthma and anaphylaxis, they also contribute to the pathophysiology of diseases in many tissues including idiopathic pulmonary fibrosis, rheumatoid disease and atherosclerosis [3], [4]. They play a central role in a mouse model of chronic asthma by inducing major pathological changes, including airway hyper-responsiveness and airway remodelling [5].

MCs possess a complex set of adhesion receptors which facilitate the migration of their progenitors from the bone marrow into various tissues where they mature and interact with various cell types and extracellular matrices (ECMs). Human MCs express a variety of adhesion receptors, including alpha2–5, alphaV, alphaM, alphaX, beta1–3 integrins, CD44, ICAM1, and cell adhesion molecule-1 (CADM1), involved in cell-ECM and cell-cell interactions [6]–[12]. Only E-cadherin is detected in human MCs by some researchers [13], [14], but not others [9], [15]. It was not found in HLMCs (Bradding, unpublished data). However, there is marked heterogeneity with respect to MC receptor expression between species, between cells in different organs, and even between cells within the same organ [16].

CADM1 is implicated in MC adhesion to fibroblasts, airway smooth muscle cells (HASMCs) and nerves [12], [17]–[19]. Adhesion of human lung MCs (HLMCs) to lung structural cells such as HASMCs and human lung fibroblasts (HLFs) and their consequent interactions have important physiological consequences on survival, proliferation, phenotypic changes and activation with mediator release of mast cells on the one hand, and augmented contractile activity and pro-fibrotic changes in HASMCs and HLFs on the other hand [3], [19]–[22].

*CADM1* belongs to a gene family of cell-cell adhesion receptors, which also include *CADM1-4*, *PVRL1-4*, *PVR*, and *CRTAM*, as annotated by Ensembl [23]. CADM1 is essential for human health and is implicated in several diseases, such as cancer, autism spectrum disorder and venous thrombosis [24]–[26]. In mice, the *CADM1* gene is implicated in cancer, radiation-induced lung fibrosis and bone structure [27]–[29]. CADM1 mediates cell-cell adhesion via interactions with counter-receptors, all of which belong to the same family [30]–[33]. In addition, CADM1 affects the localisation of other adhesion receptors, such as E-cadherin and the alpha6beta4 integrin on the cell surface of epidermal and epithelial cells, respectively [34], [35]. Similarly, other members of the *CADM* gene family are involved in recruitment of cell-cell and ECM adhesion receptors to the cell membrane, and the assembly and stabilisation of adhesion complexes [36], [37].

CADM1 is expressed as several functional isoforms in HLMCs [38], [39]. The SP4 isoform, containing exons 1–8/11–12, is the principal functional isoform in the neoplastic MC line HMC-1 [38], [39]. HLMCs also express the longer SP1 (exons 1–9/11–12) and SP6 (exons 1–12) isoforms in addition to SP4 [38], [39]. Our previous studies demonstrated differences in the survival and adhesion of mast cells overexpressing either only SP4 or mixed isoforms. SP1 plays a dominant-negative role in survival compared to SP4 [38]. The long SP1 and SP6 isoforms, expressed on the background of predominant SP4, interfere with MC adhesion to human lung fibroblasts (HLFs) or homotypic MC aggregation [19], [38], [39]. CADM1 is a major cell adhesion receptor for the adhesion of HLMCs to HASMCs and HLFs, but CADM1 blocking and downregulation do not abolish adhesion completely [12], [19]. Since both HLFs and HASMCs build ECM around cells, which provide an additional adhesion scaffold on the cell surface, ECM adhesion receptors are expected to contribute to HLMC adhesion to these cell types. Indeed, the alpha5beta1 integrin is involved in the long-term interactions of HLMCs with HASMCs [40]. Moreover, the alphaV integrin and CD44 are also implicated in the adhesion of HLMCs to HASMCs under inflammatory conditions [41].

Several lines of evidence suggest that CADM1 is involved in ErbB3/ErbB2 signal transduction and assembly of CADM1/alpha6beta4 integrin complexes on the cell membrane [35], [42]. CADM1 is essential for MC survival [20], [38], [43], and a link between CADM1 and MC survival in the absence of adhesion suggests potential CADM1-mediated constitutive signalling [38]. As an adhesion receptor, CADM1 is likely to be involved in outside-in signalling.

The CADM1 intracellular domain has FERM-binding and PDZ-binding motifs, that potentially act as a molecular scaffold for the formation of signalling complexes. The CADM1 FERM–binding site interacts with 4.1B, 4.1G and 4.1N [44]–[46]. Focal adhesion kinase p125 is another protein, which binds to the FERM–binding site of CADM1 [47]. The CADM1 PDZ-binding site interacts with a variety of PDZ proteins, including MAGUK proteins (MPP1, MPP3, MPP6 and CASK), syntenin, protein tyrosine phosphatase PTPN13 and Tiam1 [31], [42], [48]–[51]. In addition, the CADM1 extracellular domain binds the receptor tyrosine kinases ErbB2/3 and the alpha6beta4 integrin [35], [42]. Among these CADM1-binding proteins only 4.1N, 4.1G, MPP1, CASK, syntenin and FAK are expressed at the mRNA level [syntenin>MPP1>4.1N, 4.1G> CASK, FAK] in human MCs [14]. The CADM1-binding 4.1 proteins and CASK are linked to the actin cytoskeleton, providing a connection between CADM1 and the actin cytoskeleton [44], [52]. Furthermore, the receptor tyrosine kinase Kit, critical for the survival of human MCs, co-precipitates with CADM1 [12].

In summary, substantial evidence indicates that CADM1 is likely to affect the formation of membrane complexes, cell signalling and, thereby, the function of other adhesion receptors in HLMCs. We have examined this hypothesis by studying the role of CADM1 in MC adhesion to ECM, intracellular signalling and the polymerisation of filamentous actin. We show for the first time that CADM1 downregulation decreases MC adhesion to ECM and that modulation of CADM1 causes changes in surface Kit levels, tyrosine phosphorylation and the actin cytoskeleton. Our data imply that MC adhesion involves cooperating adhesion receptors which function in a stepwise manner, with a critical role for CADM1.

## Materials and Methods

### Cell Culture

The human MC line HMC-1.1 (KIT V560G) [53] was cultured in IMDM with 10% foetal bovine serum. HLMCs were isolated from healthy lung obtained at surgery for carcinoma using anti-CD117-coated Dynabeads [10]. HLMCs were cultured in DMEM supplemented with 10% FCS, and cytokines (100 ng/ml SCF, 50 ng/ml IL-6, and 10 ng/ml IL-10) as described previously [20]. Stabilised (1–2)-week old HLMCs with a final purity of >99%, were used for adenoviral transduction. HASMCs were isolated using explant culture of ASM bundles and cultured at passages 3–5 in DMEM supplemented with 10% FBS, antibiotic/antimycotic agents and non-essential amino acids as previously described [54]. They expressed ASMC-specific antigens alpha-smooth muscle actin and smooth muscle myosin with efficiency 93% and 60%, respectively. All patients gave written informed consent and the study of primary human lung mast cells and human airway smooth muscle cells was approved by the Leicestershire, Northamptonshire and Rutland Research Ethics Committee.

### Adenoviral transduction

The CADM1 cDNAs and ShRNA in adenovirus particles (BioFocus, Leiden, the Netherlands) and transduction conditions were described previously [38]. Adenoviral particles with GFP cDNA and luciferase shRNA (LucSh) were used as controls. Sh5 alone or a mix of Sh3/Sh4/Sh5, denoted here as Shm, was used for CADM1 downregulation. HMC-1 cells were transduced for 6 days. HLMCs were transduced for 4 days to prevent strong cell-cell aggregation of transduced cells which occurs at 6 days [38], thus ensuring that single cell suspensions were used in experiments.

### FACS Analysis

Cells (10^5^/vial) were washed in PBS, fixed in 4% formaldehyde in PBS and stained with 1 µg/ml chicken anti-CADM1 3E1 IgY mAb (Medical & Biological Laboratories, Japan) or IgY control, followed by Cy5-conjugated anti-IgY Ab (Millipore, UK). Surface Kit was measured using 1/50 PE-conjugated mAb YB5.B8 and control PE-conjugated IgG1 (BD Pharminogen, UK). FITC-conjugated phalloidin (Invitrogen, Life Technologies, UK) was used to quantify actin filaments (F-actin) according to the manufacturer’s recommendation. Stained cells were washed twice to remove non-specific staining. Fluorescence was examined using a FACSCanto II flow cytometer and FACSDiva2 software (BD Biosciences). Two/three biomarkers were measured simultaneously in the same samples. Geometric means were used to quantify levels of surface expression of CADM1 and Kit, and F-actin.

### Protein Analysis

Protein extracts were prepared using NP40 detergent (Invitrogen) extraction with wide spectrum inhibitors of proteases and protein phosphatases. SDS–PAGE, western blotting and protein quantification were performed as described previously [38]. When possible, blots were cut into horizontal strips with proteins of a particular range of molecular weights, probed with appropriate antibodies. Alternatively, whole blots were sequentially probed with antibodies from different species. Antibodies against CADM1 (3E1 IgY mAb, Medical & Biological Laboratories, Japan), Kit (E1, Santa Cruz Biotechnology), Kit pY823 and pY936 (44–498G and 44–500G Abs, Biosource, Life Technology, UK), phosphotyrosine (mAb pY99 sc-7020, Santa Cruz Biotechnology, Germany) and beta-actin HRP-conjugated mAb C4 (Santa Cruz Biotechnology, Germany) were used in this study.

### Mast Cell Adhesion

Adhesion assays were performed as previously described with minor modifications [10], [12], [19], [39]. HASMCs were prepared as cells from three independent donors, mixed in equal numbers. HASMCs were seeded at 10^4^ cells/well in 96 well plates in growth medium. When cells reached confluence, the medium was replaced with DMEM supplemented with insulin/transferrin/sodium selenite (ITS-3; Sigma-Aldrich, UK) for 3 days before use. HMC-1 cells, labelled with 5 µM calcein AM (Invitrogen, Life Technologies, UK) according to manufacturer’s recommendation prior to adhesion [19], [39] (10^4^ cells/0.1 ml/well), were used for adhesion to the HASMC monolayer for 30 or 60 min in the presence or absence of 5 mM EDTA. Medium and non-adherent cells were discarded. Adherent HMC-1 cells were detected by fluorescence [19], [39].

When inhibitory mAbs were used, HMC-1 cells were pre-incubated for 15 min with 10 µg/ml 9D2 IgY mAb against CADM1 (Medical & Biological Laboratories, Japan) or for 30 min with 6.5 µg/ml mAb against alpha5beta1 integrin (MAB1969, Chemicon, Millipore, UK) prior to the adhesion assay, as described previously [12], [40]. For adhesion to fibronectin (Sigma-Aldrich, UK), plates were coated with 2 µg/50 µl/well overnight and washed with PBS [40]. For adhesion to ECM, plates with HASMCs were frozen, washed on ice with a hypotonic buffer, containing 10 mM Tris pH7.5, 0.1 mM CaCl_2_, and 0.1% BSA, followed by the same buffer with 0.25% NP-40, followed by washes with PBS and blocking with 0.5% BSA in PBS on ice, as previously described [55].

### Fluorescence Microscopy

Cells were labelled as for FACS, placed on a glass slides, with or without coverslips, and analysed on an Olympus IX81 FV1000 confocal laser scanning microscope, using a 60×1.35 NA UPlanSAPO Olympus objective, image size of 1024×1024 pixels and a zoom of 3. Z-series of FITC (Ext = 488 nm, Em = 500–545 nm), PE (Ext = 559, Em = 575–620 nm) and Cy5 (Ext = 635 nm, Em = 655–755 nm) were taken in sequential scan mode with collection of transmitted light of the 488 nm scan. Images were deconvolved using Huygens Essential deconvolution software (SVI, The Netherlands). The 3D reconstructions were generated and the Z axial distance and minimal and maximal cross-sectional distances were measured using Imaris software (Bitplane, Switzerland). The colocalisation module of this software package was also used to calculate the thresholded Manders’ colocalisation coefficients M1/M2 in the colocalised volume for F-actin or Kit over CADM1.

The maximal length of actin filaments was measured as the means of four longest distances between crossed filaments and the means of four longest filaments from the cell membrane for each examined HMC-1 cell and HLMC, respectively, using ImageJ 1.46 (rsb.info.nih.gov/ij/). The means were then calculated for each group of ten transduced cells. More details are shown in Fig. S3 and Fig. S4.

### Data Analysis

Analysis was performed using GraphPad Prism 5 software. All data are presented as the mean ±SE. Differences among the groups were analysed using a one-way ANOVA, followed by the Dunnett’s test to determine whether the groups were different from a control group, or the Bonferroni’s test to compare multiple groups. Groups of data with non-parametric distribution or non-equal variances were analysed by the Kruskal-Wallis test, followed by the Dunn’s multiple comparison test. Pearson’s and Spearman’s tests were used to analyse correlations. Analysis of biomarker expression in a generalised linear model was performed using SPSS (PAWS18, IBM).

## Results

### Mast Cell Adhesion to HASM Cells and their ECM is Time-dependent

Since MC adhesion to HASMCs involves cell-cell adhesion and potentially adhesion to ECM, present on the cell surface and between the cells, we examined adhesion in conditions which permitted estimation of divalent metal cation-dependent integrin involvement. Firstly, we investigated the time-course of HMC-1 adhesion to HASMCs and their ECM. Secondly, EDTA was used to block integrins, the only ECM adhesion receptors which are dependent on divalent ions. EDTA also inhibits cadherins, but the expression of E-cadherin in human MCs is controversial (see above), and HASMCs do not express E-cadherin [56], hence cadherins are not involved in MC adhesion to HASMCs. CADM1 belongs to the family of Ca^2+^-independent cell-cell adhesion receptors, which are not affected by EDTA. The neoplastic human MC line HMC-1 was used in these experiments, since it displays a similar pattern of adhesion receptors and adhesion to those of HLMCs [12], [15], [19].

**Figure 1:**
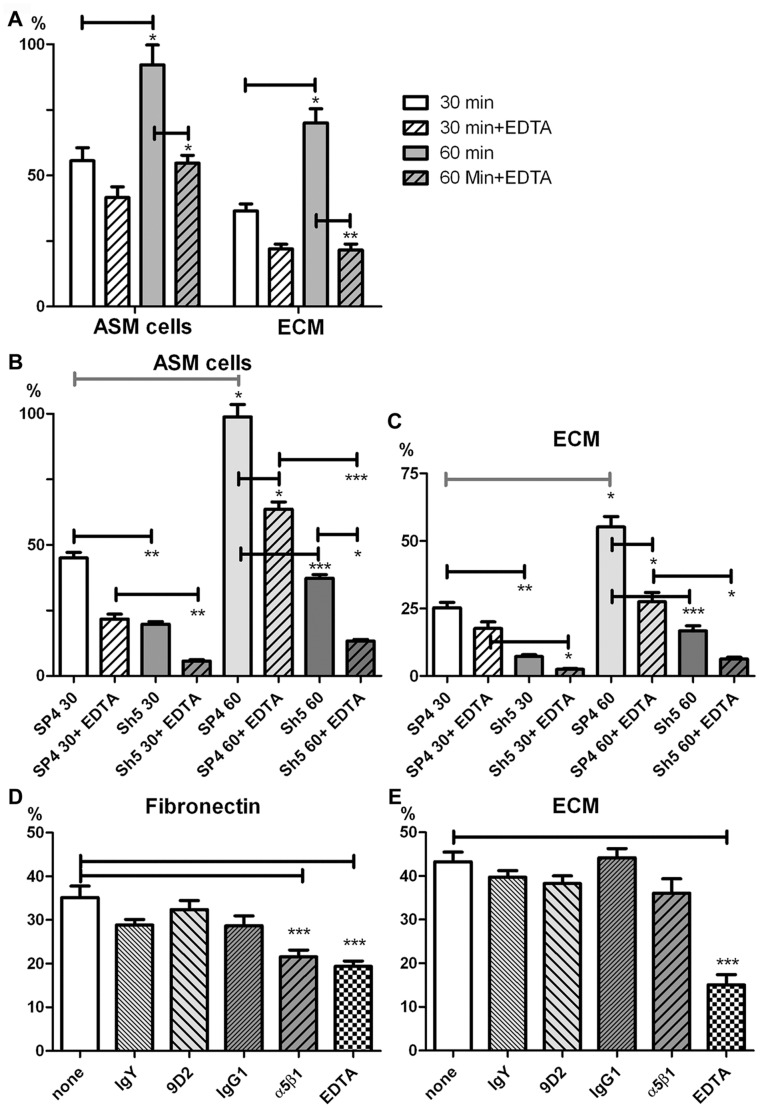
The effect of EDTA and CADM1 modulation on HMC-1 adhesion to airway smooth muscle cells and their extracellular matrices at 30 min and 60 min. **A.** HMC-1 cells (n = 2 in quadruplicate) adhered to HASMCs (n = 3) or HASMC ECM for 30 or 60 minutes in the absence or presence of 5 mM EDTA. **B and C**. HMC-1 cells (n = 2 in quadruplicate), transduced with CADM1 SP4 or shRNA (Sh5) adenoviral particles, adhered to HASMCs (n = 3 in quadruplicate)(B) or their ECM (**C**). **D and E**. HMC-1 cells (n = 2 in quadruplicate), pre-incubated with inhibitory antibodies for CADM1 (9D2) and aplha5beta1 integrin, isotype controls IgY and IgG1 or 5 mM EDTA, adhered to fibronectin (D) or HASMC ECM (E). * P<0.05; ** P<0.01; *** P<0.001.

Net adhesion of HMC-1 cells to HASMCs was 56±5% and 92±8% after 30 and 60 min, respectively (**Fig. 1A**). Thus, adhesion increased progressively over 60 min, almost reaching 100%. Adhesion of HMC-1 cells to HASMCs in the presence of EDTA was 42±4% and 55±3% after 30 and 60 min, respectively (**Fig. 1A**), indicating that integrins, inhibited by EDTA, contributed little to adhesion at 30 min, but contributed significantly to net adhesion (∼41%) after 60 min. Adhesion of HMC-1 cells to HASMC ECM was 36±3% and 70±5% after 30 and 60 min, respectively (**Fig. 1A**). EDTA reduced adhesion to ECM to 22±2% and 22±2% after 30 and 60 min, respectively (**Fig. 1A**). Again divalent cation-dependent integrins contributed more to net adhesion after 60 min (∼69%) compared to 30 min. Thus, adhesion to HASMCs appears to involve both cell-cell and cell-ECM adhesion receptors, with the latter involved mostly at an advanced stage of adhesion.

### CADM1 Regulates Adhesion of HMC-1 Cells to HASMCs and ECM

Next we examined adhesion of HMC-1 cells overexpressing the SP4 CADM1 isoform and cells with downregulated CADM1 in the presence or absence of EDTA as above. CADM1 overexpression increased surface CADM1 expression, while RNA interference reduced CADM1 expression (**Fig. S1**).

We have shown previously that CADM1 overexpression does not increase MC adhesion to HASMCs, because the binding is limited by co-receptors expressed on HASMCs, but CADM1 downregulation decreases adhesion to HASMCs [19]. Adhesion of SP4-overexpressing cells to HASMCs was 45±2% and 99±5% after 30 and 60 min, respectively, whereas adhesion of CADM1-downregulated cells (Sh5 group) in the same conditions was 20±1% and 37±1%, respectively (**Fig. 1B**). Thus, CADM1 downregulation decreased adhesion to HASMCs after 30 and 60 min, confirming a major role for CADM1 in MC adhesion to HASMCs at both initial and more advanced stages, in agreement with our previously reported data [19].

In the presence of EDTA, adhesion of SP4-overexpressing HMC-1 cells to HASMCs was reduced to 22±2% and 64±3% after 30 and 60 min, respectively; whereas adhesion of HMC-1 cells with downregulated CADM1 in the presence of EDTA was 6±1% and 13±1%, respectively (**Fig. 1B**). Thus, EDTA again reduced adhesion of CADM SP4-overexpressing cells and CADM1-downregulated cells after 60 min, suggesting integrin involvement. This integrin-dependent contribution to the net adhesion of SP4-overexpressing HMC-1 cells to HASMCs was ∼36% after 60 min. However, it contributed ∼64% to the net adhesion of HMC-1 cells with downregulated CADM1 (**Fig. 1B**). Thus, downregulation of CADM1 markedly increased the relative contribution of other adhesion receptors, e.g. divalent cation-dependent integrins, in the adhesion of HMC-1 cells to HASMCs.

Next we examined the adhesion of CADM1-modulated HMC-1 cells to the ECM of HASMCs. Adhesion of SP4-overexpressing HMC-1 cells to ECM was 25±2% and 55±4% after 30 and 60 min, respectively, whereas adhesion of CADM1-downregulated cells in the same conditions was 7±1% and 17±2%, respectively (**Fig. 1C**). EDTA further reduced ECM adhesion of SP4-overexpressing cells to 18±2% and 28±3% after 30 and 60 min, respectively, and adhesion of cells with downregulated CADM1 to 2±0% and 6±1% after 30 and 60 min (**Fig. 1C**), respectively. Importantly adhesion of SP4-overexpressing cells was always higher than adhesion of cells with downregulated CADM1 in the same conditions, despite the fact that CADM1 is not an ECM adhesion receptor.

These surprising data implied that CADM1 is involved in the ECM adhesion of HMC-1 cells either directly or indirectly. If CADM1 directly recognises specific ECM molecules, CADM1 inhibition by specific adhesion-blocking antibody would be expected to reduce adhesion to its substrate. If CADM1 does not interact with ECM directly, it may regulate ECM adhesion receptors by other mechanisms. In the latter case, CADM1-inhibitory antibody would not affect adhesion to ECM. To examine these alternative hypotheses, we studied adhesion of HMC-1 cells in the presence of CADM1-inhibitory antibody 9D2 [12] and with an alpha5beta1 integrin-inhibitory Ab, used as a negative control in adhesion to fibronectin. The alpha5beta1 integrin-inhibitory Ab and EDTA decreased HMC-1 adhesion to fibronectin from 35±3% in control to 22±2% and 19±1% respectively, but blocking CADM1 was without effect (**Fig. 1D**). Adhesion to HASMC ECM was not reduced by CADM1-blocking antibody, but was reduced by EDTA to 15±2% from 43±2% in the control (**Fig. 1E**). These data suggest that CADM1 is likely to regulate ECM adhesion indirectly, since CADM1 inhibition did not affect adhesion to ECM.

Collectively, these data indicated that 1) MC adhesion to HASMCs involves both cell and ECM adhesion receptors; 2) CADM1 is a major MC adhesion receptor providing fast initial cell-cell adhesion; 3) divalent cation-dependent integrins are major ECM adhesion receptors which contribute more significantly to MC-ECM adhesion at a more advanced stage; 4) CADM1 expression levels markedly influence the function of MC ECM adhesion receptors.

### CADM1 Influences Surface Kit Levels in Human Mast Cells

Cell adhesion via integrins requires phosphotyrosine signalling and integrin anchoring to the actin cytoskeleton [57]. Since CADM1 is linked to the actin cytoskeleton and interacts with Kit, which cross-activates integrins in MCs [58], we investigated whether CADM1 affects expression of surface Kit and assembly of actin filaments in HMC-1 cells.

**Figure 2:**
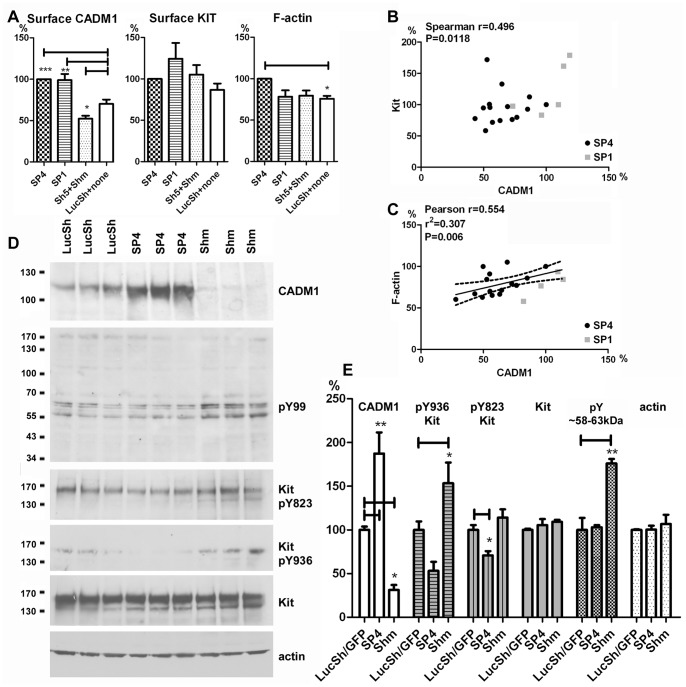
Modulated CADM1 levels in HMC-1 cells influenced surface Kit expression, an assembly of filamentous actin and tyrosine phosphorylation. **A.** HMC-1 cells were transduced with SP4, SP1, control shRNA LucSh, CADM1 shRNA (Sh5 or Shm) viral particles. Then examined for expression of surface CADM1 (total n = 30 groups from 7 transductions), surface Kit (n = 27 from 7 transductions) and amounts of F-actin (n = 23 from 4 transductions) by FACS. The control group combines LucSh-transduced and non-transduced cells, CADM1 downregulated group combines Sh5- and Shm-transduced cells. All data were expressed as a percentage of the levels in the SP-overexpressing cell group. **B and C.** Scatter plots for the data presented in **A** with correlation or regression model parameters are shown for Kit (**B**) and F-actin (**C**) as a function of CADM1. Data for SP4- and SP1-expressing cells are shown in different colours. **D.** Western blotting of protein extracts from LucSh-, SP4- and Shm-transduced HMC-1 cells, with 3 independent transductions for each group, developed with Abs shown on the right of the blots. **E.** Protein bands, shown in **D** and Fig. S**2C**, were quantified. Bands in SP4 (n = 5) and Shm (n = 4) groups were expressed as percentages of control LucSh/GFP group (n = 5) for each protein, except phosphotyrosine 58–63 kDa bands (n = 3). * P<0.05; ** P<0.01; *** P<0.001.

Surface CADM1 was significantly modulated by SP4 and SP1 overexpression or RNA interference in HMC-1 cells, but no statistically significant changes were detected in the surface Kit levels or F-actin (**Fig. S1**). To increase statistical power for these analyses, control groups (LucSh shRNA and non-transduced cells) and CADM1-downregulated groups (Sh5 and Shm) were combined. The total dataset was normalised to either the SP4 group (a larger dataset, **Fig. 2**) or the control group (a smaller dataset, **Fig. S2**). Surface CADM1 was increased in SP4- and SP1-overexpressing cells to 138±11% and 146±3%, respectively, compared to the combined control group (100%), whereas RNA interference reduced CADM1 to 72±3% (**Fig. S2A**).

Kit levels on the cell surface slightly varied among the groups of transduced cells, but no statistical differences were found (**Fig. 2A** **, Fig. S2A**). However, when we examined the potential relationship between surface expression levels of these proteins, surface Kit levels correlated positively with modulated CADM1 levels (**Fig. 2B**). The larger dataset for Kit (**Fig. 2B**), which included both SP1 and SP4 overexpression, displayed a non-parametric distribution and could not be analysed further. Since HMC-1 cells express predominantly one functional isoform SP4, all groups with modulated CADM1, apart from the SP1 group, expressed only this isoform. The data points for SP1 were clustered towards one end (**Fig. 2B**). The smaller dataset also showed that surface Kit was strongly associated with changes in surface CADM1 (**Fig. S2B**). CADM1 was responsible for ∼46% of surface Kit (R^2^ = 0.46) in HMC-1 cells (**Fig. S2B**).

### CADM1 Influences Actin Polymerisation in Human Mast Cells

F-actin was increased in SP4-overexpressing cells compared to the control group (**Fig. 2**, **Fig. S2A**). F-actin strongly correlated with modulated CADM1 (**Fig. 2C**). Regression analysis of the larger dataset for CADM1 and F-actin showed that CADM1 is responsible for 31% of F-actin assembly (r^2^ = 0.307) in HMC-1 cells (**Fig. 2C**). When we examined the data for SP1 and SP4 isoforms they were again distinct as shown in the graph in **Fig. 2C**. A linear regression model predicted that both CADM1 (a numerical variable) and isoforms SP4 or SP1 (a categorical variable) had a major effect on F-actin (P = 0.000 for CADM1 and P = 0.001 for isoforms). The smaller dataset showed a trend of a correlation between F-actin and CADM1 (P = 0.089, **Fig. S2B**).

### CADM1 Expression Influences Tyrosine Phosphorylation in Human Mast Cells

Since modulation of CADM1 induced changes in surface Kit levels, Kit-mediated tyrosine phosphorylation was likely to be modulated as well. Therefore, we examined phosphotyrosine signalling in cells with CADM1 either overexpressed or downregulated. In these experiments, total CADM1 was increased to 187±24% in the SP4-overexpressing group and reduced to 31±6% in the CADM1-downregulated Shm group compared to the LucSh control (**Fig. 2D–2E** **, Fig. S2C**). Conversely, modulation of surface CADM1 was smaller than modulation of total CADM1 in agreement with our previous data [38], indicating that CADM1 delivery to the cell surface is likely to be regulated. CADM1 modulation did not influence total levels of Kit or beta-actin (**Fig. 2D–2E** **, Fig. S2C**) in agreement with our previous results [38]. CADM1 SP4 overexpression reduced phosphorylation of Kit Tyr-823 in the kinase active site to 71±5%, whereas CADM1 downregulation increased autophosphorylation of Tyr-936 to 153±24% (**Fig. 2D–2E** **, Fig. S2C**). CADM1 downregulation also increased tyrosine phosphorylation of several protein bands with molecular weights ∼58–63 kDa to176±5% (**Fig. 2D–2E**).

In summary, these data show that CADM1 in HMC-1 contributes to 1) docking of Kit to the cell surface, 2) the amounts of F-actin and 3) tyrosine phosphorylation of Kit and other proteins.

### CADM1 Regulates Actin Cytoskeleton and Cell Signalling in Human Lung Mast Cell Cells

**Figure 3:**
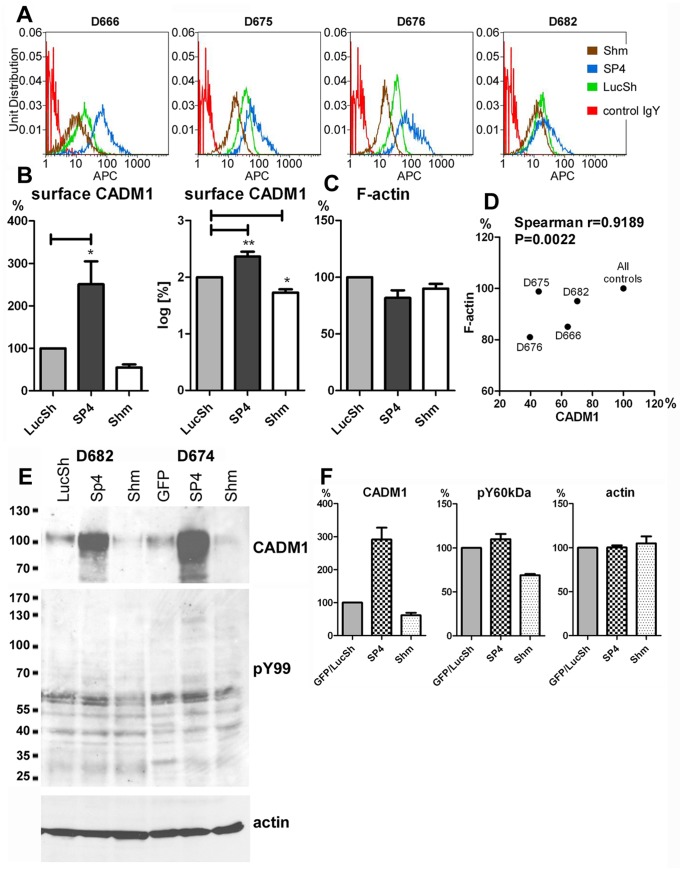
Downregulation of CADM1 in human lung mast cells reduced the assembly of filamentous actin and tyrosine phosphorylation. **A.** HLMCs from various donors were transduced with SP4, control shRNA (LucSh) or CADM1 shRNA (Shm) viral particles, followed by fluorescent staining of surface CADM1 and F-actin. Surface CADM1 expression is shown in histograms. **B.** CADM1 in the SP4 and Shm groups was expressed as percentages of the control LucSh group and quantified as percentage and log-transformed percentage. (n = 5 for LucSh and SP4, n = 4 for Shm). These data were presented in Fig. 1 in [19]. **C.** HLMCs with modulated CADM1 were also co-stained for F-actin. The data are shown in the graph. Correlation between surface CADM1 and F-actin in LucSh- or Shm-transduced HLMCs is shown in a scatter plot with correlation parameters. **E.** Western blotting of protein extracts from LucSh-, SP4- and Shm-transduced HLMCs from D682 and D674, developed with Abs shown on the right. The protein bands were quantified and the data are shown in the graph.

Next we examined whether CADM1 modulation affects the amount of F-actin and cell signalling in HLMCs. Surface CADM1 was overexpressed to 251±54% or downregulated to 55±7% in several populations of HLMCs (**Fig. 3A–3B**), as described previously [19]. Modulation of CADM1 did not significantly change levels of F-actin (**Fig. 3C**), but F-actin was reduced in all four HLMC populations with reduced CADM1 and there was a correlation between downregulated CADM1 and F-actin (**Fig. 3D**). In two populations of HLMCs, CADM1 downregulation decreased tyrosine phosphorylation of 58–60 kDa proteins (**Fig. 3E–3F**). Since Kit phosphorylation is not detectable in non-stimulated HLMCs, it was not studied here. Thus, we have evidence of CADM1-mediated effects on the actin cytoskeleton and tyrosine phosphorylation in both HLMCs and the human mast cell line HMC-1.

### CADM1 Downregulation Influences Cell Shape and Increases the Length of F-actin Filaments in Human Mast Cells

**Figure 4:**
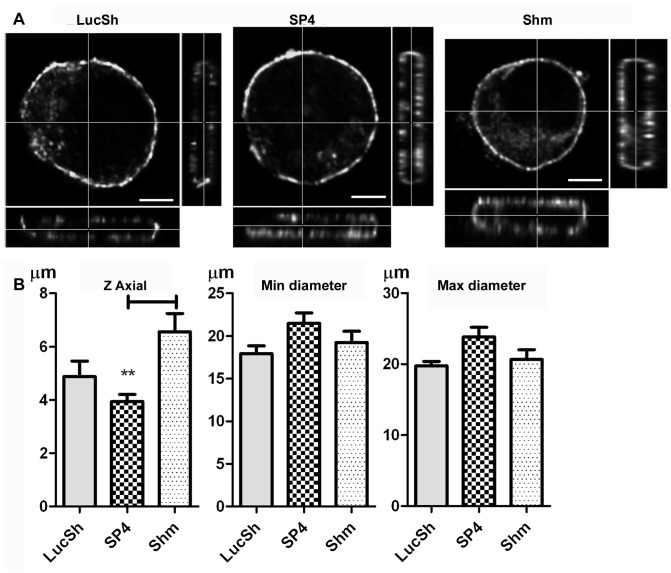
CADM1 downregulation in HMC-1 cells influenced the cell shape. **A.** LucSh-, SP4- and Shm-transduced HMC-1 cells from an experiment shown in Fig. **2D**, were co-stained for CADM1 and examined by confocal laser scanning microscopy. Glass slides with HMC-1 cells in suspension were covered with coverslips. The 3D reconstructions were generated to show the XY cross-section through the cell in the large panel, the YZ overview in the right panel and XZ overview in the bottom panel for each image. Bar is 5 µm. **B.** The Z axial distance and minimal and maximal cross-sectional (X and Y) distances were measured and analysed for LucSh- (n = 11), SP4- (n = 14) and Shm-transduced (n = 14) cells. ** P<0.01.

To examine the consequences of CADM1-dependent modulation of F-actin assembly and surface Kit, HMC-1 cells co-stained for surface CADM1, surface Kit and F-actin were examined microscopically. Cells in suspension between glass-slides and coverslips had the shape of crumpets (**Fig. 4A**, **Video S1, S2, S3**). The horizontal cross-sectional measurements were similar in all transduced groups, but the cell height (Z-axial) measurements were higher in the cells with downregulated CADM1 (6.6±0.7 µm) compared to those in the SP4-overexpressing cells (3.9±0.3 µm), whereas there was no difference between the LucSh control cells (4.9±0.6 µm) and SP4-overexpressing cells (**Fig. 4B**).

**Figure 5:**
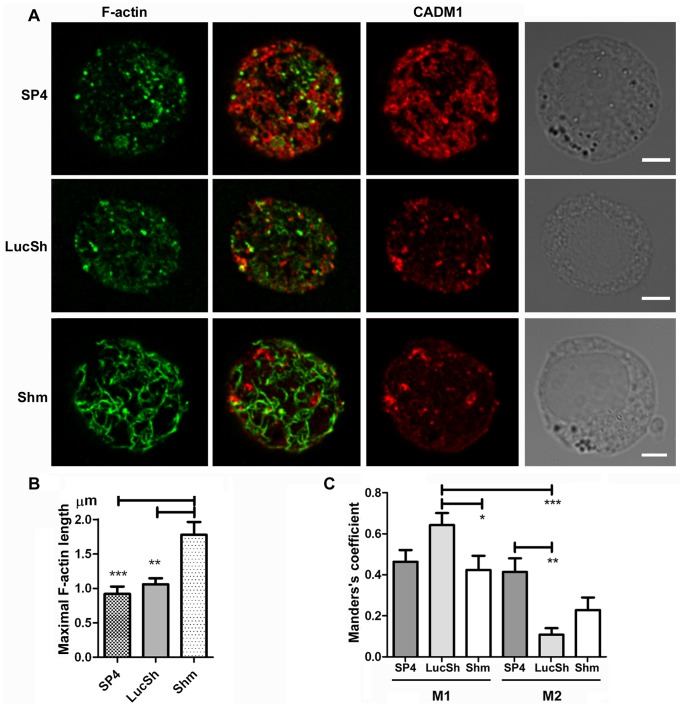
CADM1 downregulation in HMC-1 cells increased the length of cortical actin filaments. LucSh-, SP4- and Shm-transduced HMC-1 cells from an experiment shown in Fig. **2C** and **3A**, stained for F-actin and CADM1, were examined by confocal laser scanning microscopy. The same optical section of the cell surface (equivalent to the side of a crumpet) is shown for each protein. F-actin and CADM1 are shown in false colours. The right column shows light-transmission images. Bar is 5 µm. **B.** The maximal length of actin filaments was calculated as the means of 4 longest distances between crossed filaments for each examined HMC-1 cell, then the data for n = (10–11) cells for each group were analysed. ** P<0.01; *** P<0.001. **C.** Graphs are shown for the thresholded Manders’s coefficients M1 for colocalisation of F-actin with CADM1 and M2 for colocalisation of CADM1 with F-actin in the SP4, LucSh and Shm-groups of cells (n = 7, n = 7 and n = 6 cells with two surfaces each, respectively). * P<0.05, ** P<0.01; *** P<0.001.

Next we studied cortical actin filaments in HMC-1 cells with modulated CADM1. Cortical F-actin in cells with downregulated CADM1 looked strikingly different compared to the control LucSh-transduced cells or SP4-overexpressing cells (**Fig. 5A**, **Fig. S3**, **Video S1, S2, S3**). F-actin filaments appeared as numerous short points on the cell surface in the SP4-overexpressing and control cells, whereas they were a network of sparse, widely separated long filaments in cells with downregulated CADM1. The maximal length of F-actin filaments was significantly higher in the CADM1 downregulated group (1.78±0.19 µm) compared to that in the SP4 and LucSh groups (0.92±0.11 µm and 1.06±0.09 µm, respectively, **Fig. 5B**).

In order to estimate colocalisation of CADM1 with F-actin and Kit, we measured colocalisation coefficients (for details see [59]–[61]). The thresholded Manders’ coefficients M1 and M2 are often interpreted as co-occurrence of measured biomarkers [61]. Surprisingly, there was little colocalisation between CADM1 and F-actin in merged images (**Fig. 5A**, **Video S1, S2, S3**). The thresholded Manders’s coefficient M1 for colocalisation of F-actin with CADM1 was 0.46±0.06, 0.64±0.06 and 0.42±0.07 for the SP4, LucSh and Shm groups of cells, respectively (**Fig. 5C**), indicating medium colocalisation of the F-actin signal with CADM1, particularly increased in control LucSh cells. The Manders’s coefficient M2 for colocalisation of CADM1 with F-actin was 0.41±0.07, 0.11±0.03 and 0.23±0.06 for the SP4, LucSh and Shm groups of cells, respectively (**Fig. 5C**). It was significantly increased in the SP4 group compared with control LucSh cells. Colocalisation of F-actin with CADM1 (M1) was higher than colocalisation of CADM1 with F-actin (M2, **Fig. 5C**).

**Figure 6:**
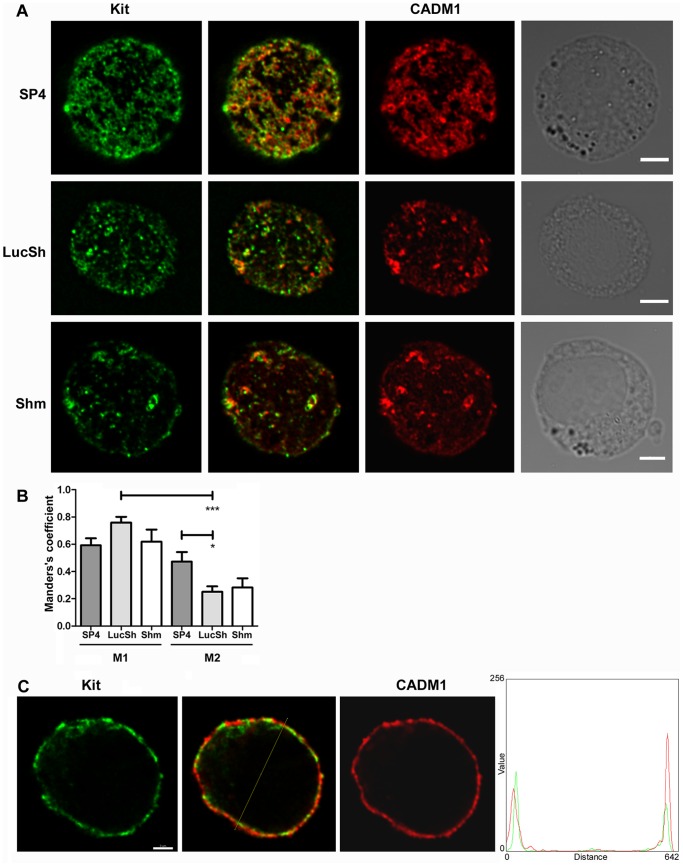
Modulated CADM1 levels in HMC-1 cells influenced Kit. LucSh-, SP4- and Shm-transduced HMC-1 cells from an experiment shown in Fig. **2C** and **3A**, stained for surface Kit and CADM1, were examined by confocal laser scanning microscopy. The same optical section of the cell surface (equivalent to the side of a crumpet) is shown for each protein. Kit and CADM1 are shown in false colours. The right column shows light-transmission images. Bar is 5 µm. **B.** Graphs are shown for the thresholded Manders’s coefficients M1 for colocalisation of Kit with CADM1 and M2 for colocalisation of CADM1 with Kit in the SP4, LucSh and Shm-groups of cells (n = 7, n = 7 and n = 6 cells with two surfaces each, respectively). * P<0.05, *** P<0.001.**C.** The middle optical section is shown for the LucSh-transduced HMC-1 cell shown in A. The line through the dual stained cell was scanned and the intensity of staining is presented in a graph on the right.

There was colocalisation between Kit and CADM1 on the cell surface in merged images (**Fig. 6A, 6C**) in agreement with previously published data [62]. The thresholded Manders’ coefficient M1 for colocalisation of Kit with CADM1 was reasonably high: 0.59±0.05, 0.76±0.04 and 0.62±0.09 in SP4-overexpressing, LucSh- and Shm-transduced cells respectively (**Fig. 6B**), indicating that more than 50% of the Kit signal in these optical sections colocalised with CADM1. Manders’s coefficient M2 for colocalisation of CADM1 with Kit was 0.47±0.07, 0.25±0.04 and 0.28±0.07 in SP4-overexpressing, LucSh- and Shm-transduced cells, respectively (**Fig. 6B**). Colocalisation of Kit with CADM1 was therefore higher than colocalisation of CADM1 with Kit in control cells. Colocalisation of F-actin or Kit with CADM1 (M1) was higher than colocalisation of CADM1 with F-actin or Kit (M2) in control cells (**Fig. 6B**).

**Figure 7:**
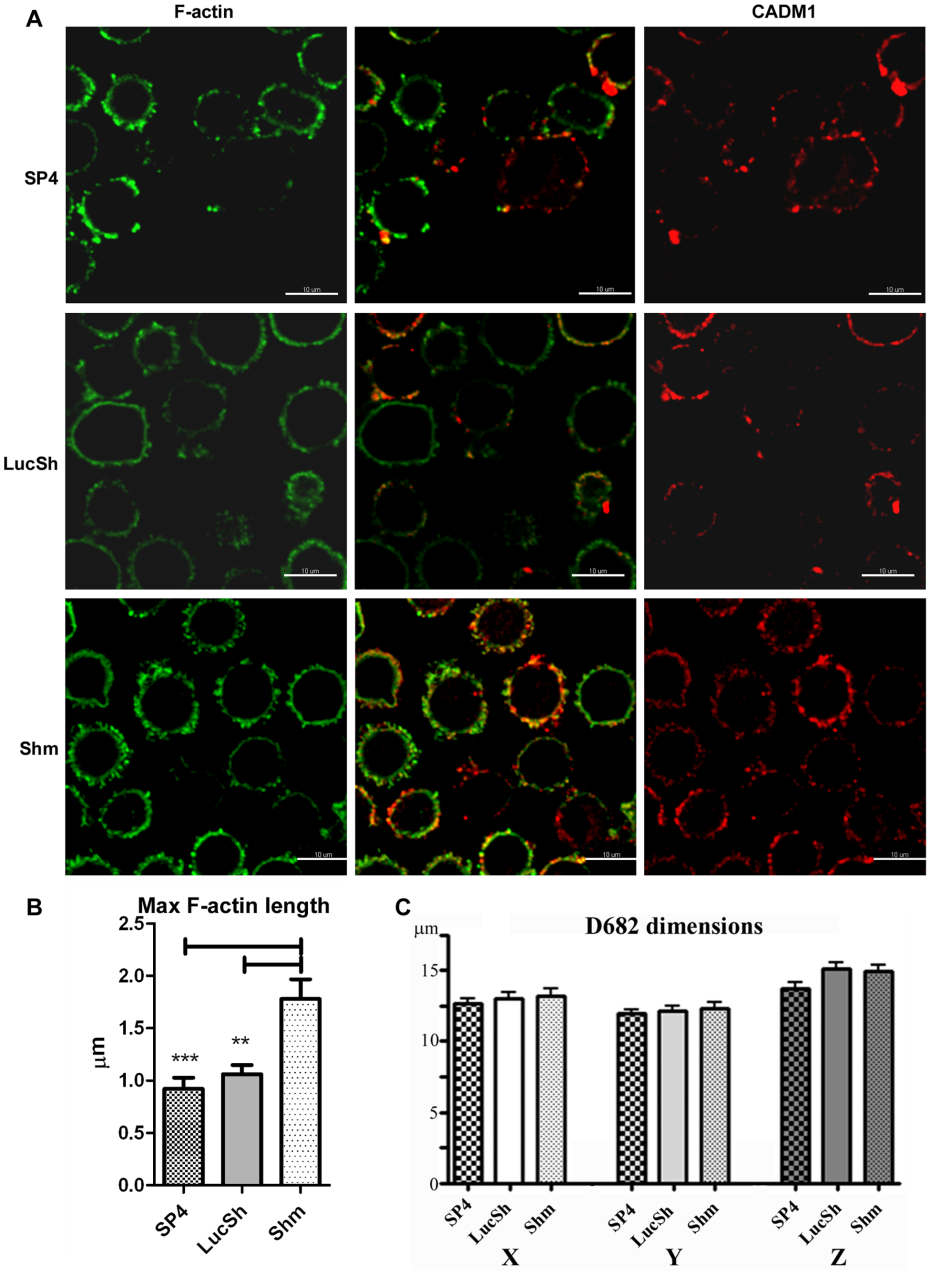
CADM1 Downregulation in human lung mast cells increased the length of cortical actin filaments. LucSh-, SP4- and Shm-transduced HLMCs D682 from an experiment shown in Fig. **3A** and **3E** were stained for F-actin and surface CADM1. Glass slides with HLMCs in suspension were examined by confocal laser scanning microscopy. The same optical section of the cell surface is shown for each protein. F-actin and CADM1 are shown in false colours. Bar is 10 µm. **B.** The maximal length of actin filaments was measured as the means of 4 longest filaments on the cell membrane for each examined cell, then the data for all groups of transduced cells were analysed (n = 10 cell for each group). **, P<0.01; ***, P<0.001. **C.** The maximal and minimal cross-sectional (X, Y) and Z axial distances were measured and analysed for LucSh- (n = 13), SP4- (n = 9) and Shm-transduced (n = 15) cells.

Next we examined HLMCs from donor D682 with modulated CADM1 microscopically. This time cells were in suspension on glass slides without coverslips. Again cortical F-actin looked strikingly different in cells with downregulated CADM1 (**Fig. 7A** **, Fig. S4**). The maximal length of F-actin filaments was significantly higher in the CADM1 downregulation group (2.07±0.08 µm) compared to that in the SP4 and LucSh groups (1.34±0.04 µm and 1.27±0.05 µm, respectively, **Fig. 7B**). Thus, the cortical actin filaments were shorter in controls and SP4-overexpressing cells than in cells with reduced CADM1 in both neoplastic HMC-1 cells and HLMCs. Similar to HMC-1 cells, some limited co-localisation of CADM1 with actin filaments could be seen as a yellow colour particularly in control cells and cells with reduced CADM1 (**Fig. 7A**). The HLMCs had round shapes, but no differences between the transduced groups were observed in cell dimensions (**Fig. 7C**).

## Discussion

Cell adhesion is a complex process and involves various adhesion receptors; it is also influenced by growth factor receptor tyrosine kinases [57], [63]. Current models of adhesion involve cooperation between ECM adhesion receptors integrins and syndecans [57]. Cell-cell adhesion receptors nectins (*PVRL*) and CD155 (*PVR*) of the *CADM* gene family recruit integrins and cadherins to the cell membrane and promote the assembly and stabilisation of adhesion complexes [36], [37]. MCs express a complex set of adhesion receptors, including ECM adhesion receptors (CD44, integrins) and cell-cell adhesion receptors (CADM1, ICAM-1) [6]–[11], [17]. Activation of the receptor tyrosine kinase Kit is known to increase integrin engagement in MCs, whereas Kit inhibition reduces it [58], [64], [65].

Here we show for the first time that CADM1 plays a key role in the regulation of divalent cation-dependent integrin receptors, contributing to MC adhesion to ECM. We also show for the first time that CADM1-promotes Kit docking to the cell surface and polymerisation of cortical F-actin in MCs and affects tyrosine signalling. All these factors are critical for integrin function and are likely to play roles in CADM1-mediated adhesion.

In this study we have confirmed that adhesion of human MCs to HASMCs involves CADM1, in agreement with previously published data [12], [19]. We also show for the first time that MC adhesion to HASMCs involves adhesion to their ECM. We found that CADM1 provides adhesion at both 30 and 60 min, but the contribution of divalent ion-dependent integrins to ECM adhesion becomes relatively greater at 60 min compare to 30 min. This is in agreement with time-course data on integrin-dependent MC adhesion to vitronectin and fibronectin, which reaches maximal values after 1 h [66], [67]. The role of other ECM receptors which are not dependent on divalent ions, such as CD44, appears to be relatively small in human MC adhesion to HASMCs and their ECM in our experimental conditions.

Here we demonstrate that these adhesion receptors work stepwise: CADM1 was responsible for both initial adhesion and more advanced adhesion to HASMCs, whereas integrins contributed to adhesion mostly at an advanced stage. Unexpectedly downregulation of CADM1 resulted in reduced ECM adhesion. No ECM proteins have been identified among CADM1-interacting proteins to-date (see http://www.ncbi.nlm.nih.gov/gene/23705) [68]. Inhibition of CADM1 with the adhesion-blocking mAb 9D2 did not affect MC adhesion to either ECM or fibronectin, suggesting that it is the CADM1 levels, not CADM1 binding, which are important. Hence, CADM1 is unlikely to bind to ECM directly (although not impossible and requires further investigation), but it is more likely to regulate ECM adhesion receptors in MCs indirectly.

Interactions between CADM1 family receptors and other adhesion receptors have been described previously [36], [37]. CADM1 is known to interact specifically with the hemidesmosomal alpha6beta4 integrin, but not alpha5beta1 or alpha6beta1 integrins, in colorectal adenocarcinoma Caco-2 cells [35]. In contrast to our results, CADM1 modulation in those cells did not affect adhesion to ECM substrates laminin-332 or matrigel [35]. Human MCs express predominantly beta1 integrins and do not express alpha6beta4 integrin [14]. Therefore, we studied whether CADM1 affects ECM adhesion via other mechanisms, such as tyrosine signalling and actin polymerisation, which are essential for integrin activation and anchoring, respectively. We also hypothesised that CADM1 may influence activity of other adhesion receptors via interactions with Kit on the cell surface.

To investigate this, we examined whether CADM1 modulation would affect cortical filamentous actin and surface Kit levels using FACS analysis. In neoplastic HMC-1 cells, the levels of surface Kit and F-actin displayed linear dependence on CADM1 levels. CADM1 was responsible for a half of surface Kit and a third of the F-actin levels in these cells. Moreover, CADM1 levels markedly influenced tyrosine phosphorylation in HMC-1 cells. CADM1 upregulation reduced tyrosine-823 phosphorylation in the kinase domain of Kit, suggesting downregulation of Kit activity. In contrast, CADM1 downregulation augmented autophosphorylation of the tyrosine-936, indicating increased kinase activity. In addition, CADM1 downregulation markedly increased tyrosine phosphorylation of protein bands 58–63 kDa. Some of them are likely to be SRC family kinases Lyn (59 kDa) and Fyn (61 kDa), which bind to activated Kit [69] and become more tyrosine phosphorylated upon recruitment. Similarly, we found some evidence of reduced levels of actin polymerisation and tyrosine phosphorylation in two populations of HLMCs with downregulated CADM1.

Altogether, CADM1 upregulation increased surface Kit and reduced its kinase activity and, vice versa, CADM1 downregulation decreased surface Kit and increased its activation in HMC-1 cells that have mutated Kit V560G. Since CADM1 and Kit co-precipitate [20], it is possible that CADM1 physically interacts with a particular inactive conformation of Kit, and as a result, increased CADM1 levels would stabilise Kit in this conformation on the cell surface. Conversely, decreased CADM1 would not stabilise inactive Kit; thus, mutated Kit would dimerise and autophosphorylate C-terminal tyrosines, this would result in increased internalisation of Kit and removal from the cell surface. This potential mechanism requires further investigation, because it may have functional implications for the pathophysiology of leukaemia and pulmonary fibrosis. Interestingly, high levels CADM1 in mixed-lineage leukaemia-rearranged acute monoblastic leukaemia provide better overall survival [70]. Expression of Kit was also reported in this disease [71]. Recent data suggest that lung fibroblasts express increased levels of membranous SCF, the Kit ligand, in pulmonary fibrosis [22]. Their co-culture with HLMCs results in increased activation and proliferation of both cell types [22], driving the progression of disease.

Although a connection between CADM1 and F-actin was proposed some time ago [44], we did not find published data demonstrating the reorganisation of the actin cytoskeleton by CADM1. We show that polymerisation of actin i) positively correlates with CADM1 levels, ii) depends on the expressed isoforms SP4 or SP1 and iii) affects the length of cortical actin filaments in human mast cells. We have shown previously that SP4 and SP1 isoforms are functionally different in regard to mast cell survival and expression of anti-apoptotic protein MCL-1 [38], homotypic mast cell adhesion [38], and mast cell adhesion to human lung fibroblasts [19], [39]. This study shows that these isoforms also affect F-actin assembly differentially. Since the SP4 and SP1 isoforms differ only by 11 amino acids in the extracellular domain, their effects on intracellular events may be a result of their differential shedding, thus leading to altered cell signalling for SP1 compared to SP4.

CADM1-induced modulation of the actin cytoskeleton determined its flexibility in HMC-1 cells flattened between glass slides and coverslips. Interestingly, CADM1 downregulation also resulted in a flat morphology of epithelial HEK293 cells and impaired filopodia formation in neurons [47], [51]. Since CADM1 is responsible only for ∼30% of F-actin and is connected to the actin cytoskeleton indirectly via linker proteins, it is feasible that these proteins organise local points for actin polymerisation with actin filaments extending outwards along the cell membrane; this would explain the relatively limited colocalisation of CADM1 and F-actin.

Functional links between proteins necessitate their localisation in the same cell compartments. Since CADM1 was responsible for a substantial portion of surface Kit and F-actin levels, we expected to find partial colocalisation of them with CADM1. The Manders’s coefficient M1 for colocalisation of surface Kit with CADM1 was higher than M1 for colocalisation of F-actin with CADM1 in all groups of HMC-1 cells; thus it was consistent with FACS data indicating that CADM1 is responsible for a half of Kit and only a third of cortical F-actin on the cell surface. In contrast, in the control LucSh-transduced cells the Manders’ coefficients M2 for colocalisation of CADM1 with Kit or F-actin were lower than the corresponding M1 coefficients for colocalisation of these proteins with CADM1, indicating that CADM1 participates in formation of multiple other protein complexes on the cell surface. Both data from microscopy (Manders’s coefficient) and FACS suggest some functional relationships between CADM1 and Kit/F-actin; therefore, further studies are required to elucidate their interactions at a molecular level.

Re-arrangement of the actin cytoskeleton affects various cell functions. In MCs it may influence two critical processes: migration and secretion. An ability to change cell shape is important for MCs in order to i) migrate from bone marrow into specific tissue sites through multiple layers of various cells and matrices, and ii) redistribute to the key positions in infection or diseases. MCs with reduced CADM1 are likely to migrate with less efficiency, because they have lower adhesion capacity and a more rigid cell shape. MCs are specialised secretory cells and degranulation involves extensive cytoskeleton reorganisation [72]. Therefore, rearrangement of actin filaments in MCs with reduced CADM1 may affect secretion. The role of CADM1 in these specialised MC functions requires future studies.

**Figure 8:**
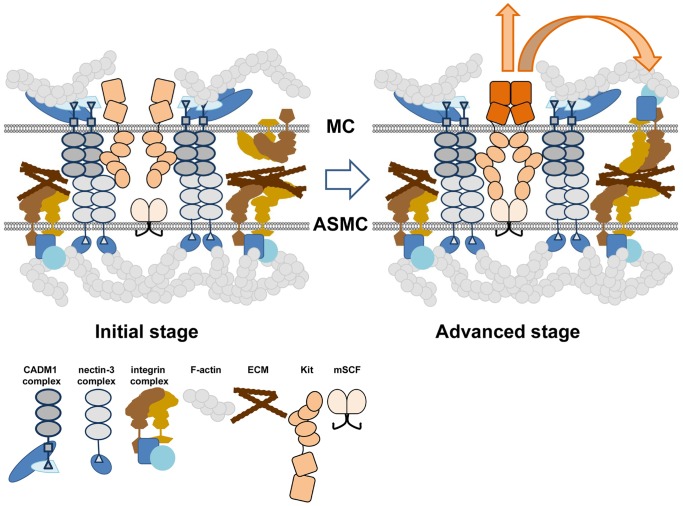
A two-step model of mast cell adhesion. A diagram illustrates cell membrane complexes of MC and ASMC at the top and bottom, respectively. In MCs, CADM1 dimer forms a complex with 4.1 proteins and CASK, bound to F-actin. In ASMCs, nectin-3 dimer forms a complex with afadin, connected to F-actin [73]. In an initial stage of MC adhesion to ASMCs, CADM1 dimers interact with nectin-3 dimers on the surface of ASMCs [19]. This places inactive integrins in close proximity to their ECM ligands on ASMC surface. Kit, stabilised by CADM1, becomes close to membranous CSF in ASMCs. During an advanced adhesion stage, active Kit dimers bind to mCSF and initiate signal transduction to integrins in MCs. Integrins and other ECM receptors on MCs find their ECM ligands and interact with them. Activated integrins bind talin, vinculin and other structural proteins, which link integrins to cortical F-actin [57], [74], organised by CADM1.

Collectively our data show for the first time that CADM1 is not only a major human MC adhesion receptor involved in cell-cell and cell-ECM adhesion, but it also controls the assembly of actin filaments and docks Kit at the cell surface. On this basis we propose a stepwise model of MC adhesion, which includes interactions between various receptors and Kit (**Fig. 8**). According to this model, CADM1 provides initial adhesion between human MCs and HASMCs and, as a result, ECM receptors are positioned in close proximity to ECM on the surface of HASMCs, thereby facilitating the adhesive interaction of other adhesion receptors. Integrin engagement is also facilitated by quick anchoring of integrin-mediated adhesion complexes to the actin cytoskeleton, which is partly dependent on CADM1. Furthermore, surface Kit stabilised by CADM1, binds membranous SCF on HASMCs, becomes activated and initiates phosphotyrosine signalling to cross-activate ECM adhesion receptors. Whether activated Kit is bound to CADM1 at this stage needs further examination. Overall, CADM1 influences several parameters in cells which facilitate cell adhesion through other receptors.

It is becoming increasingly obvious that cell adhesion involves a variety of cooperating adhesion receptors and growth factor receptor tyrosine kinases [63]. CADM1 is not just one of them, it is a receptor which is likely to organise other adhesion receptors and signalling complexes at the MC membrane. Therefore, future studies should elucidate the exact structure of CADM1-binding complexes and interactions between them in order to understand cross-talk between various adhesion receptors and links to Kit-mediated phosphotyrosine signalling in mast and other cell types.

## Supporting Information

Figure S1 **Modulated CADM1 levels in HMC-1 cells.** HMC-1 cells were transduced with SP4, SP1, control shRNA (LucSh), CADM1 shRNA (Sh5 or Shm) viral particles and then examined for expression of surface CADM1, surface Kit and F-actin. None stands for non-transduced cells. The data of 3–7 transductions were normalised to expression in SP4 group (A) or LucSh group (B). The size of datasets for each protein is shown in Fig. 2 and Fig. S2. * P<0.05; ** P<0.01.
(TIF)

Figure S2 **Modulated CADM1 levels in HMC-1 cells influenced Kit phosphorylation. A.** HMC-1 cells were transduced with SP4, SP1, LucSh, Sh5 or Shm viral particles and then examined for surface expression of CADM1 (total n = 36 for all groups from 7 transductions) and Kit (n = 23 from 5 transductions), and amounts of F-actin (n = 17 from 3 transductions) by FACS. All data were expressed as a percentage of the levels in the control LucSh+non-transduced group. **B.** Scatter plots for the data presented in **A** with regression model parameters for Kit and F-actin as a function of CADM1. Data for SP4 and SP1 are shown in different colours. *, P<0.05; ***, P<0.001. **C.** Western blotting of protein extracts from LucSh-, GFP-, SP4- and Sh5-transduced HMC-1 cells (2 independent transductions) developed with Abs shown on the right.
(TIF)

Figure S3 **CADM1 downregulation in HMC-1 cells increased the length of cortical actin filaments.** SP4- and Shm-transduced HMC-1 cells, stained for F-actin (central panel) from an experiment shown in Fig. **5**, were examined by confocal laser scanning microscopy. The left panel shows the same optical section for light-transmission images. Several measurements of the longest actin filaments, equivalent to longest distances between crossed filaments, are shown on the photographs. The length in micrometres is shown on the right of the figure. The four highest measurements (highlighted in grey) were used to calculate the average maximal length of actin filaments for each examined HMC-1 cell.
(TIF)

Figure S4 **CADM1 downregulation in HLMCs increased the length of cortical actin filaments.** SP4- and Shm-transduced HLMC population from donor D682 HMC-1 cells, stained for F-actin (central panel) from an experiment shown in Fig. **7**, were examined by confocal laser scanning microscopy. The left panel shows the same optical section for light-transmission images. Measurements of the longest actin filaments for 5 cells in SP4- and Shm-transduced cell populations, respectively, are shown on the photographs. The length in micrometres is shown on the right of the figure. The four highest measurements for each cell were used to calculate the average maximal length of actin filaments for each examined cell. Black dots in the left panel are metal beads used for mast cell isolation.
(TIF)

Video S1 **CADM1 and filamentous actin on the cell surface of HMC-1 cell with overexpressed SP4 CADM1.** SP4-transduced HMC-1 cell, stained for surface CADM1 (purple) and F-actin (green), were examined by confocal laser scanning microscopy. Images were deconvolved using Huygens Essential deconvolution software and 3D reconstructions prepared in Imaris software using the surface-rendering option. Surface transparency allows to see areas of colocalisation, indicated by changed colour.
(ZIP)

Video S2 **CADM1 and filamentous actin on the cell surface of control HMC-1 cell.** LucSh-transduced HMC-1 cell, stained for surface CADM1 (purple) and F-actin (green), were examined by confocal laser scanning microscopy as described above.
(ZIP)

Video S3 **CADM1 and filamentous actin on the cell surface of HMC-1 cell with downregulated CADM1.** Shm-transduced HMC-1 cell, stained for surface CADM1 (purple) and F-actin (green), were examined by confocal laser scanning microscopy as described above.
(ZIP)
