# Correction: CADM1 Controls Actin Cytoskeleton Assembly and Regulates Extracellular Matrix Adhesion in Human Mast Cells

**DOI:** 10.1371/journal.pone.0091600

**Published:** 2014-02-28

**Authors:** The PLOS ONE Staff

## Notice of Republication

This article was republished on February 5, 2014, to include Supplementary Videos S2 and S3, the links to and the labels and legends for which were incorrectly omitted from the original online version of the article. The original PDF is unchanged.
