# Supplementary figures and images for: CADM1 Controls Actin Cytoskeleton Assembly and Regulates Extracellular Matrix Adhesion in Human Mast Cells
Source: PLoS One. 2014 Jan 22;9(1):e85980. doi: 10.1371/journal.pone.0085980 (PMC3899107; doi:10.1371/journal.pone.0085980)

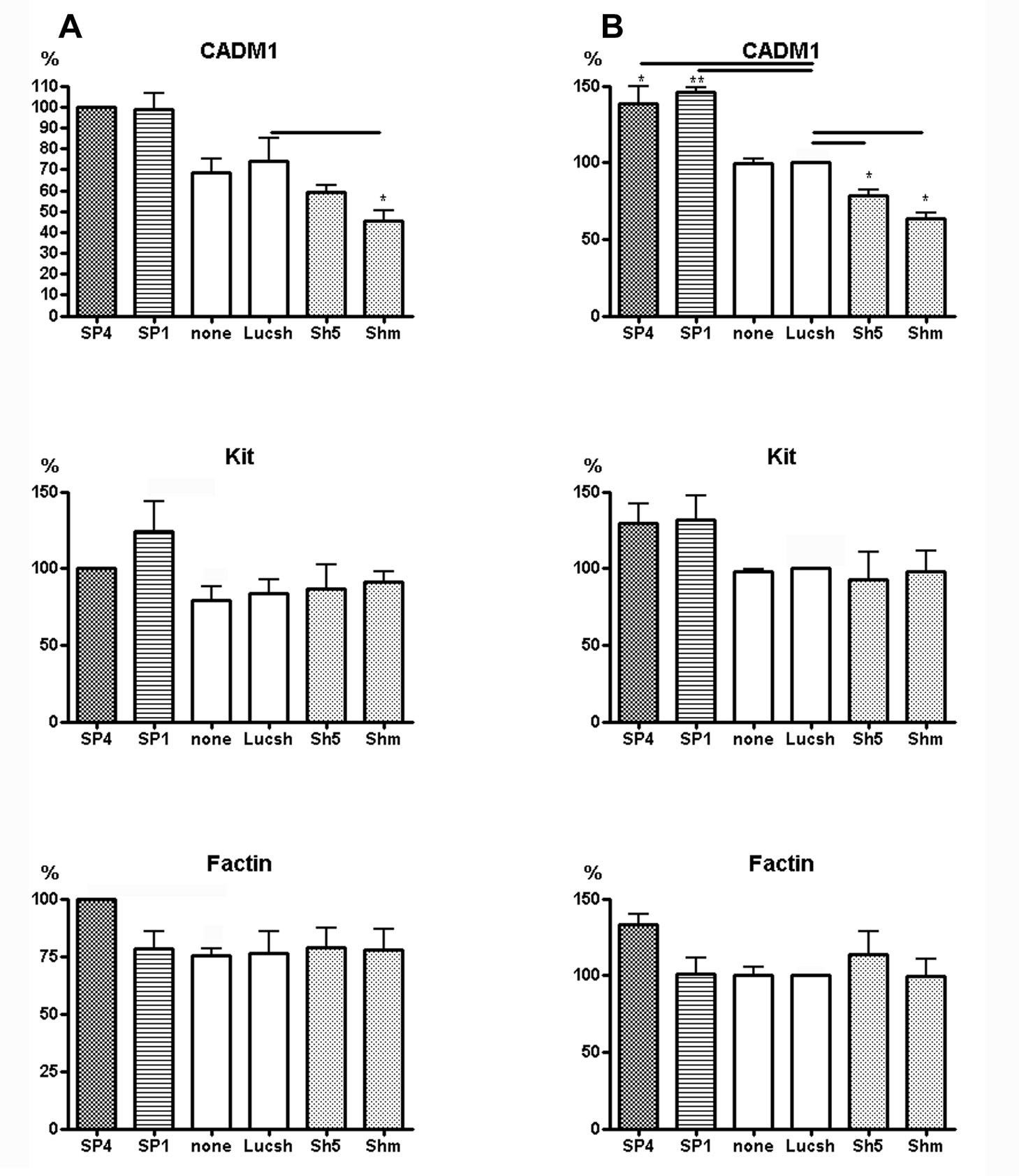

Supplement: Figure S1 — Modulated CADM1 levels in HMC-1 cells. HMC-1 cells were transduced with SP4, SP1, control shRNA (LucSh), CADM1 shRNA (Sh5 or Shm) viral particles and then examined for expression of surface CADM1, surface Kit and F-actin. None stands for non-transduced cells. The data of 3–7 transductions were normalised to expression in SP4 group (A) or LucSh group (B). The size of datasets for each protein is shown in Fig. 2 and Fig. S2. * P<0.05; ** P<0.01. (TIF) [file pone.0085980.s001.tif]

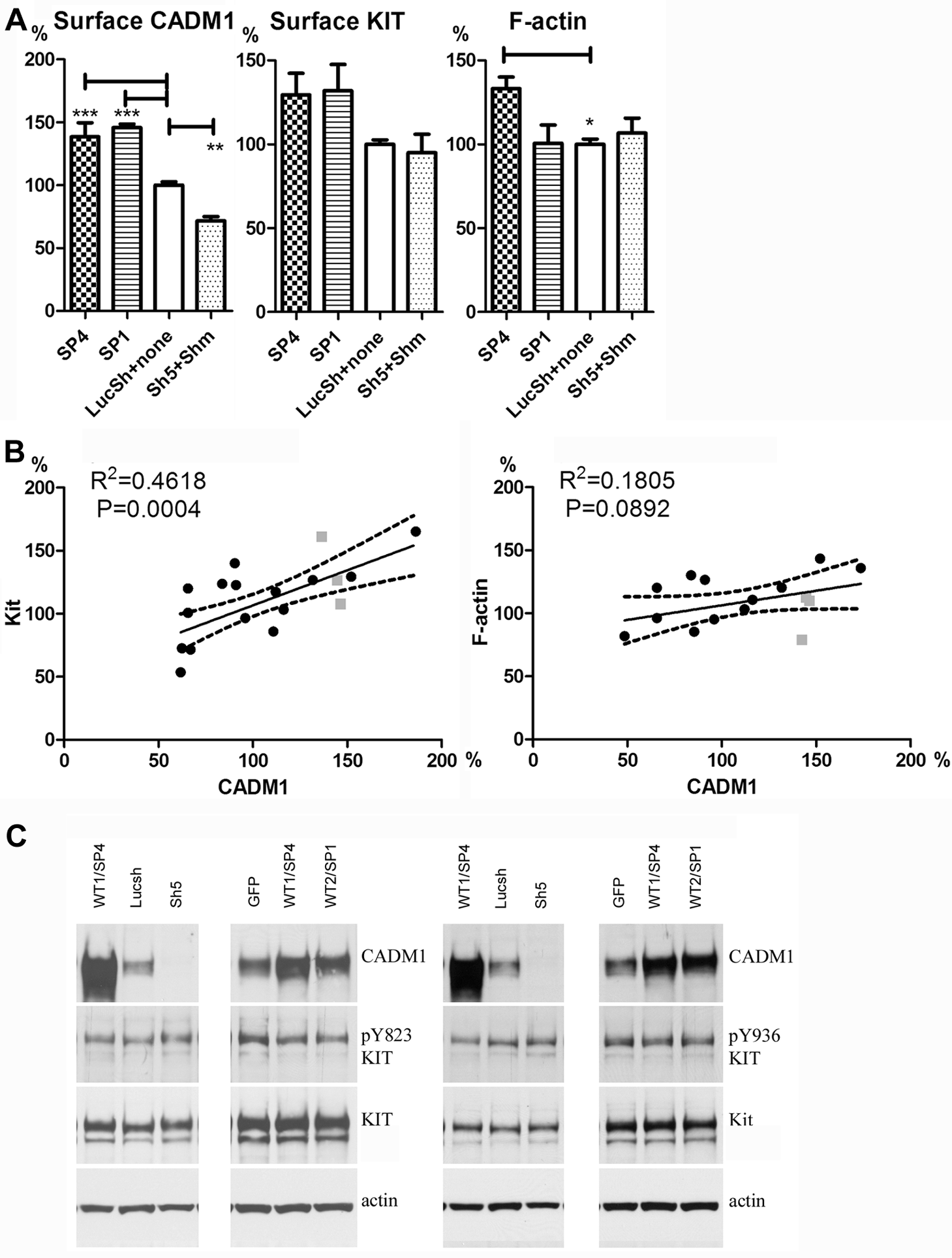

Supplement: Figure S2 — Modulated CADM1 levels in HMC-1 cells influenced Kit phosphorylation. A. HMC-1 cells were transduced with SP4, SP1, LucSh, Sh5 or Shm viral particles and then examined for surface expression of CADM1 (total n = 36 for all groups from 7 transductions) and Kit (n = 23 from 5 transductions), and amounts of F-actin (n = 17 from 3 transductions) by FACS. All data were expressed as a percentage of the levels in the control LucSh+non-transduced group. B. Scatter plots for the data presented in A with regression model parameters for Kit and F-actin as a function of CADM1. Data for SP4 and SP1 are shown in different colours. *, P<0.05; ***, P<0.001. C. Western blotting of protein extracts from LucSh-, GFP-, SP4- and Sh5-transduced HMC-1 cells (2 independent transductions) developed with Abs shown on the right. (TIF) [file pone.0085980.s002.tif]

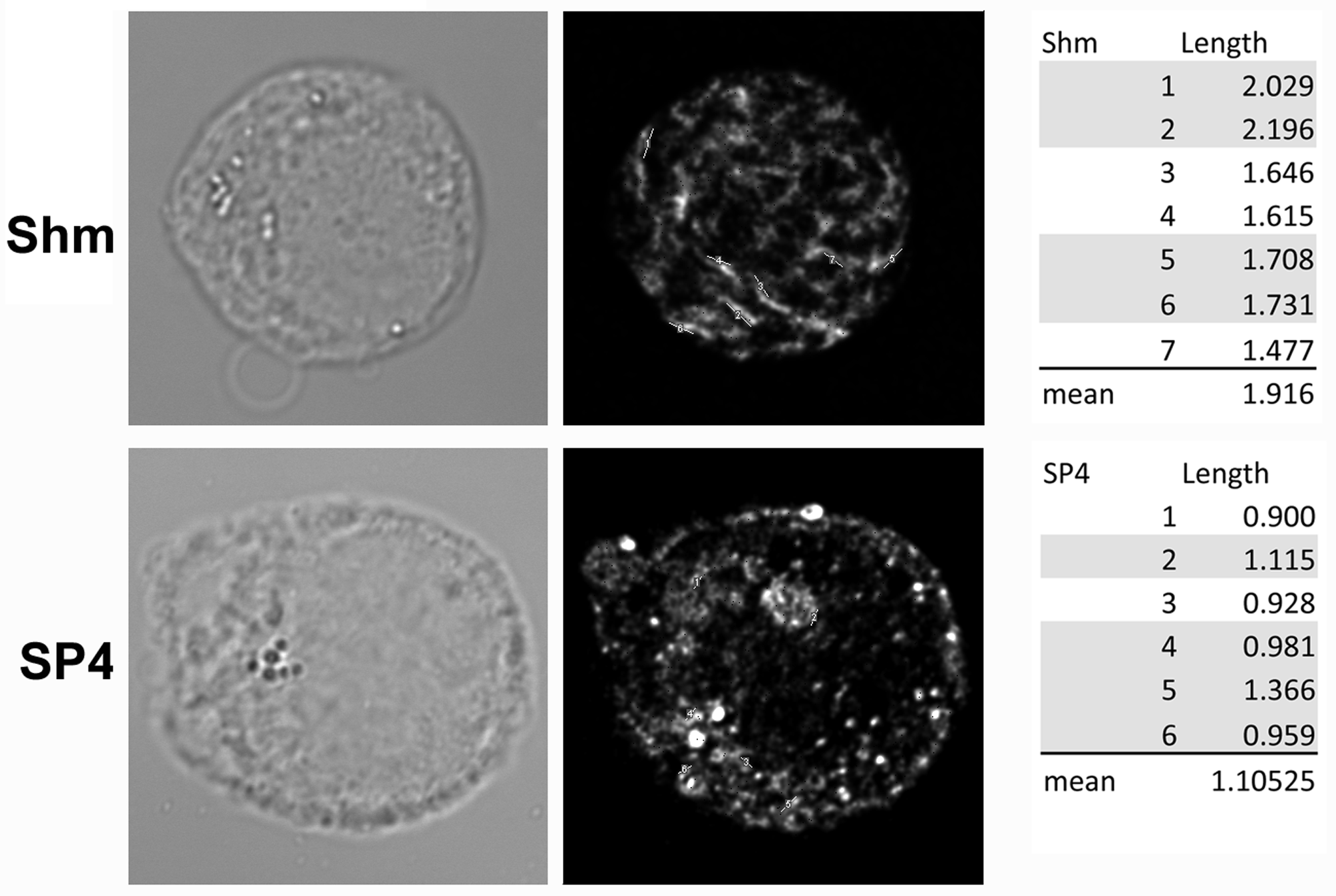

Supplement: Figure S3 — CADM1 downregulation in HMC-1 cells increased the length of cortical actin filaments. SP4- and Shm-transduced HMC-1 cells, stained for F-actin (central panel) from an experiment shown in Fig. 5 , were examined by confocal laser scanning microscopy. The left panel shows the same optical section for light-transmission images. Several measurements of the longest actin filaments, equivalent to longest distances between crossed filaments, are shown on the photographs. The length in micrometres is shown on the right of the figure. The four highest measurements (highlighted in grey) were used to calculate the average maximal length of actin filaments for each examined HMC-1 cell. (TIF) [file pone.0085980.s003.tif]

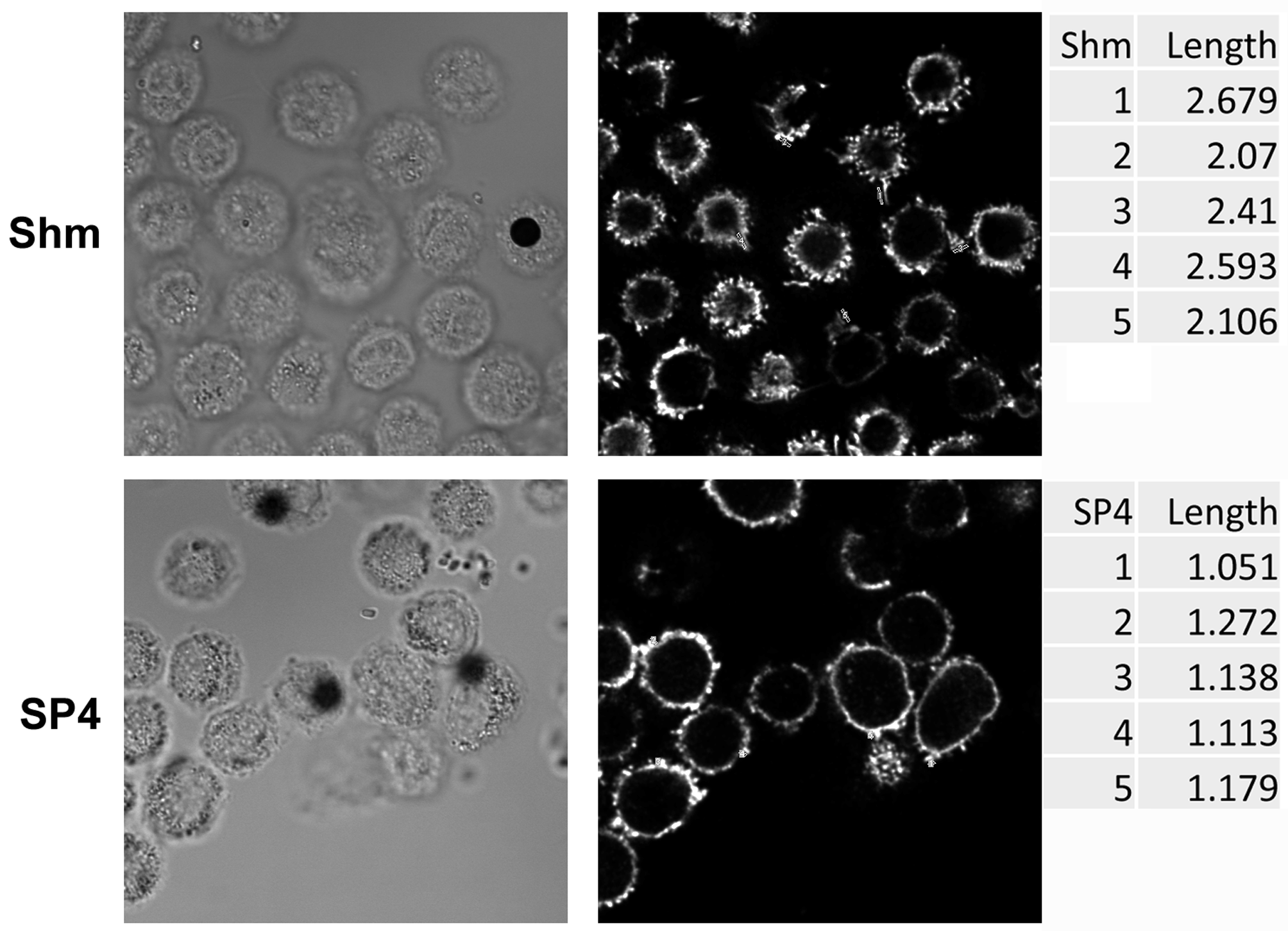

Supplement: Figure S4 — CADM1 downregulation in HLMCs increased the length of cortical actin filaments. SP4- and Shm-transduced HLMC population from donor D682 HMC-1 cells, stained for F-actin (central panel) from an experiment shown in Fig. 7 , were examined by confocal laser scanning microscopy. The left panel shows the same optical section for light-transmission images. Measurements of the longest actin filaments for 5 cells in SP4- and Shm-transduced cell populations, respectively, are shown on the photographs. The length in micrometres is shown on the right of the figure. The four highest measurements for each cell were used to calculate the average maximal length of actin filaments for each examined cell. Black dots in the left panel are metal beads used for mast cell isolation. (TIF) [file pone.0085980.s004.tif]
